# Characterization of *Pinus densiflora* var*. zhangwuensis* S.J.Zhang, C.X.Li & X.Y.Yuan complete chloroplast genome

**DOI:** 10.1080/23802359.2021.2005486

**Published:** 2021-11-29

**Authors:** Qijing Xia, Chunying Hao, Baixi Zhang, Yousry A. El-Kassaby, Wei Li

**Affiliations:** a National Engineering Laboratory for Forest Tree Breeding, College of Biological Sciences and Technology, Beijing Forestry University, Beijing, China; b Liaoning Research Institute of Sandy Land Control and Utilization, Fuxin City, China; c Department of Forest and Conservation Sciences, Faculty of Forestry, University of British Columbia, Vancouver, Canada

**Keywords:** *Pinus densiflora* var*. zhangwuensis* S.J.Zhang, C.X.Li & X.Y.Yuan, Pinaceae, chloroplast genome, phylogenetic relationship

## Abstract

*Pinus densiflora* var*. zhangwuensis* S.J.Zhang, C.X.Li & X.Y.Yuan, a tree species with promising afforestation potential in northern China. Here, we assembled and annotated the complete chloroplast (cp) genome of *P. densiflora* var*. zhangwuensis* using the Oxford Nanopore sequencing technology. The cp genome was 119,725 bp in length, exhibiting a typical quadripartite structure with a large single-copy (LSC: 65,552 bp) and a small single-copy (SSC: 53,183 bp) separated by a pair of inverted repeats regions (IRA and IRB: each of 495 bp) region. The overall GC content is 37.3%. The genome was predicted to encode 112 distinct genes, including 72 protein-coding, 36 tRNA, and four rRNA genes. Maximum-likelihood (ML) phylogenetic for cp genome sequences of 18 Pinaceae species revealed that *P. densiflora* var*. zhangwuensis* was closely related to *Pinus sylvestris.*

In 1990, Pinus densiflora var. zhangwuensis S.J.Zhang, C.X.Li & X.Y.Yuan was first discovered in a *Pinus sylvestris* plantation in Zhanggutai Township, Fuxin City, Liaoning Province, China. Compared with *P. sylvestris*, *P. densiflora* var*. zhangwuensis* grows faster, and has stronger cold, drought, and salt-alkali resistance (Meng et al. 2010). More interestingly, *P. densiflora* var*. zhangwuensis* exhibits strong resistance to *Sphaeropsis sapinea* which causes serious harm to *Pinus* species in several countries covering northern and southern hamispheres (Swart and Wingfield 1991). Advances in seed orchards’ designs are expected to deliver crops with maximum seed yield and genetic gains (Yang et al. 2020), which undoubtedly will benefit the improvement of *P. densiflora* var*. zhangwuensis* seed setting rate. At present, *P. densiflora* var*. zhangwuensis* research is mainly focused on the construction of genetic map (Lei et al. 2004), understanding the physiological-biochemical characteristics. Among these genomic endeavors, research on *P. densiflora* var*. zhangwuensis* origin is paramount for enhancing the species breeding and conservation programs (Yan et al. 1999). The characterization of *P. densiflora* var*. zhangwuensis* chloroplast (cp) genome is a powerful tool for unraveling the species phylogeny and enhancing intended intra-specifc hybridization activities. Here, we utilized the Oxford Nanopore sequencing technology to completely sequence and annotate *P. densiflora* var*. zhangwuensis* chloroplast genome and analyzed the resulting sequence along with other 17 pines species and *Taxus baccata* as outgroup to determine their phelogentic relationships.

Leaf specimens from *P. densiflora* var*. zhangwuensis* trees growing in a plantation in the Zhanggutai Township, Fuxin City, Liaoning Province, China (42°07′N, 121°53′E) were collected and were stored in the Beijing Advanced Innovation Center For Tree Breeding by Molecular Design, Beijing Forestry University under voucher number 376099. *P. densiflora* var*. zhangwuensis* cpDNA was extracted by using a modified CTAB method (Song et al. 2011) and sequenced by using the Oxford Nanopore sequencing technology to capitalize on its long-read advantages (Cao et al 2016). The resulting cpDNA whole genome was assembled by Flye v2.8.3 (https://github.com/fenderglass/Flye), annotated in CPGAVAS2 v2 (http://47.96.249.172:16019/analyzer/annotate) (Shi et al. 2019), and the complete open reading frame (ORF) was predicted according to the input sequence using TBtools v0.665 (https://github.com/CJ-Chen/TBtools) (Chen et al. 2020). The cpDNA sequences of *P. densiflora* var*. zhangwuensis* along with 17 published pineaceae plastomes and *Taxus baccata* as outgroup were compared by using MAFFT v7.037 (Katoh and Standley 2013). TVM + F + G4 substitution model in IQ-tree v1.6.12 (Lam-Tung et al. 2015) was used to infer the Maximum-likelihood (ML) phylogenetic tree with 1000 bootstrap repeats (Li and Guo 2006).

The results indicated that the cpDNA genome of *P. densiflora* var*. zhangwuensis* (GenBank accession number MZ677091) has a typical quadruple structure with a large single copy (LSC: 65,552 bp) and a small single copy (SSC: 53,183 bp) separated by a pair of inverted repeats regions (IRA and IRB: each of 495 bp). The overall GC content is 37.3%. The genome was predicted to encode 112 distinct genes, including 72 protein-coding, 36 tRNA, and four rRNA genes. ML phylogenetic for cpDNA genome sequences of the 18 Pinaceae species revealed that *P. densiflora* var*. zhangwuensis* was closely related to *P. sylvestris* (Figure 1).

**Figure 1.**
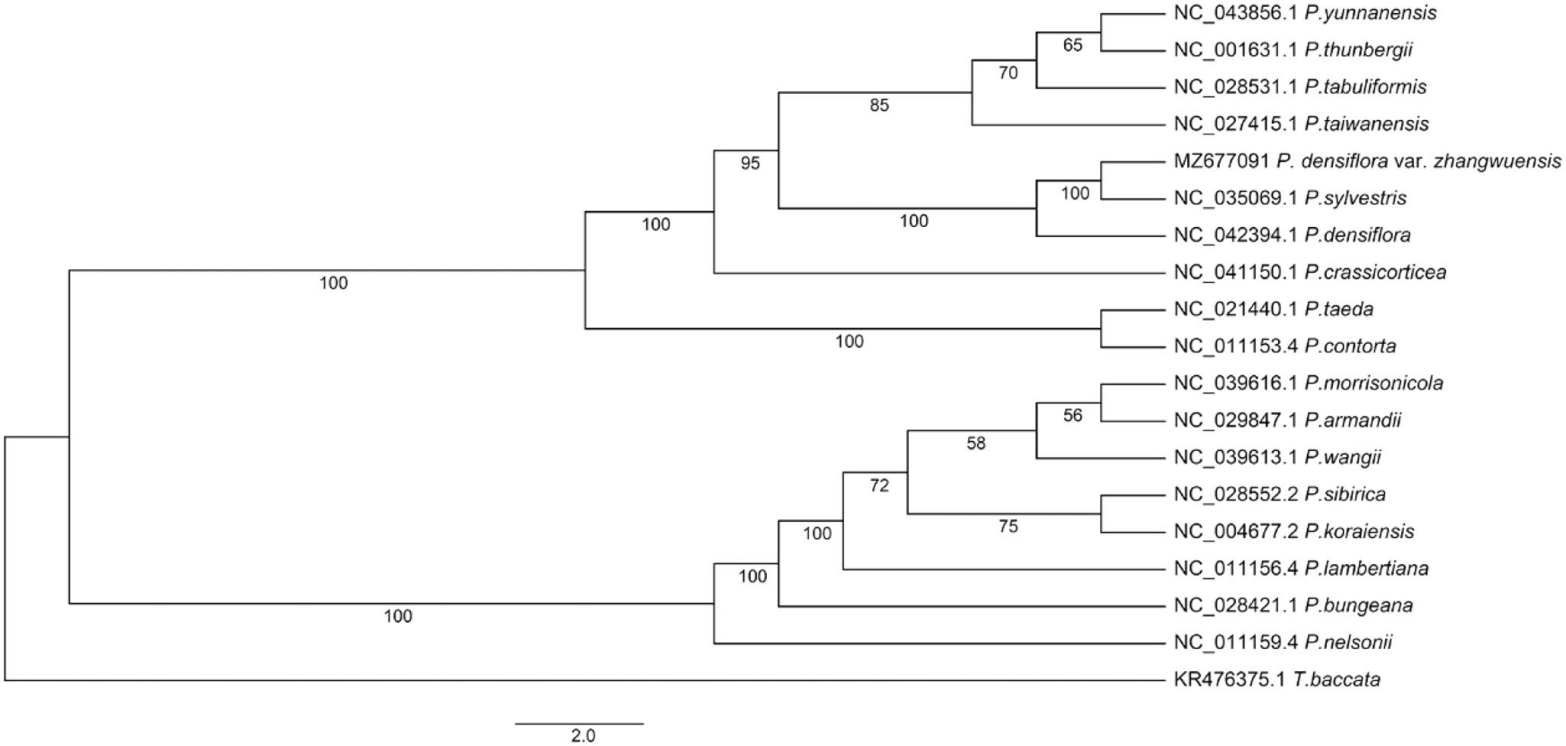
Maximum-likelihood phylogenetic tree based on complete chloroplast genome sequences of *P.densiflora* var*. zhangwuensis* and 17 other Pinaceae species (note: *Taxus baccata* was used as outgroup). Bootstrap support values are labeled at each node.

## Data Availability

The genome sequence data are available in GenBank of NCBI at (https://www.ncbi.nlm.nih.gov/) under the accession no. MZ677091. The associated BioProject, SRA, and Bio-Sample numbers are PRJNA751417, SRR15371579 and SAMN20518691 respectively.

## References

[CIT0001] Cao C-X, Han W, Zhang H-P. 2016. Application of third generation sequencing technology to microbial research. Microbiol China. 43(10):2269–2276.

[CIT0002] Chen C, Chen H, Zhang Y, Thomas H-R, Frank M-H, He Y, Xia R. 2020. Tbtools: an integrative toolkit developed for interactive analyses of big biological data. Mol Plant. 13(8):1194–1202. 32585190 10.1016/j.molp.2020.06.009

[CIT0003] Katoh K, Standley D-M. 2013. MAFFT multiple sequence alignment software version 7: improvements in performance and usability. Mol Biol Evol. 30(4):772–780. 23329690 10.1093/molbev/mst010 PMC3603318

[CIT0004] Lam-Tung N, Schmidt H-A, Arndt V-H, Quang M-B. 2015. IQ-TREE: a fast and effective stochastic algorithm for estimating maximum-likelihood phylogenies. Mol Biol Evol. 32(1):268–274. 25371430 10.1093/molbev/msu300 PMC4271533

[CIT0005] Lei Z-Y, Zhou F-Y, Wang M, Zheng J-M. 2004. On origin of *Pinus densiflora* var*. zhangwuensis*. J Beihua Univ (Nat Sci). 5(2):169–175.

[CIT0006] Li J-F, Guo M-Z. 2006. A review of phylogenetic tree reconstruction technology. Acta Electron Sin. 34(11):2047–2052.

[CIT0007] Meng P, Li Y-L, Zhang B-X, Zhang X-L, Lei Z-Y, Song X-D. 2010. A comparative study on physiological characteristics of drought resistance of *Pinus densiflora* var*. zhangwuensis* and *Pinus. sylvestris* var*. mongolica* in Sandy Soil. Sci Silvae Sin. 46(12):56–63.

[CIT0008] Shi L-C, Chen H-M, Jiang M, Wang L-Q, Wu X, Huang L-F, Liu C. 2019. CPGAVAS2, an integrated plastome sequence annotator and analyzer. Nucleic Acids Res. 47(W1):W65–W73. 10.1093/nar/gkz345 PMC6602467 31066451

[CIT0009] Song Y-B, Wu G-L, Niu H-B. 2011. Study on application of the modified CTAB method in the extraction of genomic DNA from walnut leaves. J Shanxi Agric Univ (Nat Sci Ed). 31(2):109–112.

[CIT0010] Swart WJ, Wingfield MJ. 1991. Biology and control of *Sphaeropsis sapinea* on *Pinus* species in South Africa. Plant Dis. 75(8):761–766.

[CIT0011] Yan C, Lei Z, Xu Z. 1999. Analysis of the origin and the taxonomic position of using RAPD markers. Sci Silvae Sin. 35(4):25–30.

[CIT0012] Yang B-N, Sun H-H, Qi J-D, Niu S-H, A.Ei-Kassaby Y, Li W. 2020. Improved genetic distance-based spatial deployment can effectively minimize inbreeding in seed orchard. For Ecosyst. 7(1):117–127.

